# A Case of Failed Dual Antiplatelet Therapy With Oral Anticoagulant in the Prevention of Acute Ischemic Stroke

**DOI:** 10.7759/cureus.15915

**Published:** 2021-06-25

**Authors:** Oluyemisi Amoda, Elmarie A, Ese Uwagbale

**Affiliations:** 1 Internal Medicine, Brookdale University Hospital Medical Center, Brooklyn, USA

**Keywords:** dual antiplatelet, rivaroxaban, anticoagulant, therapy, acute, cerebrovascular accident, ischemic, stroke, atrial fibrillation, aspirin

## Abstract

Cerebrovascular accidents (CVA) or strokes cause focal neurological deficits, which may be due to rupture or occlusion of blood vessels supplying areas of the brain. Atrial fibrillation (AF) is an independent and significant risk factor for ischemic CVA, mainly via the embolic pathway. This is evident as newly diagnosed patients with AF are typically started on anticoagulation therapy if their CHA2DS2-VASc score is equal to or greater than two. Furthermore, ischemic CVA may occur as a thrombotic phenomenon due to significant vessel atherosclerotic disease involving plaque ulceration and rupture. Such a phenomenon may be curtailed with antiplatelet therapy in at-risk patient populations, particularly as a form of secondary prevention. This case highlights the unfortunate incidence of an ischemic CVA in a patient using dual antiplatelet therapy (DAPT) and anticoagulation.

## Introduction

Cerebrovascular accidents (CVA), commonly known as strokes, are caused by a lack of blood flow to some parts of the brain, causing focal neurological deficits, coma, or even resulting in death. The lack of blood flow can either be from rupture or occlusion of a vessel supplying brain areas. Approximately 795,000 people suffer from strokes every year, of which 87% of the strokes are ischemic [1]. It is commonly associated with cardiovascular risk factors such as hypertension, atrial fibrillation (AF), peripheral vascular disease, coronary artery disease, hyperlipidemia, and diabetes mellitus. Because AF is a major cause of ischemic stroke, a pivotal step in the management of AF is anticoagulation to prevent the occurrence of a CVA. In addition, patients with high cardiovascular risks are typically started on some form of an antiplatelet to prevent an ischemic event.

## Case presentation

Our patient is a 93-year-old female with a medical history significant for AF, peripheral vascular disease, hypertension, hyperlipidemia, and a previous cerebrovascular accident. She lived with her son and took atorvastatin, clopidogrel, ezetimibe, and aspirin for her peripheral vascular disease; losartan and nifedipine for hypertension; metoprolol succinate and rivaroxaban for AF. She had been compliant with her medications, as confirmed by her and her son. However, she presented to the Emergency Department (ED) with a one- to two-hour history of difficulty with speech and right-sided weakness, which she had developed while having breakfast. 

**Figure 1:**
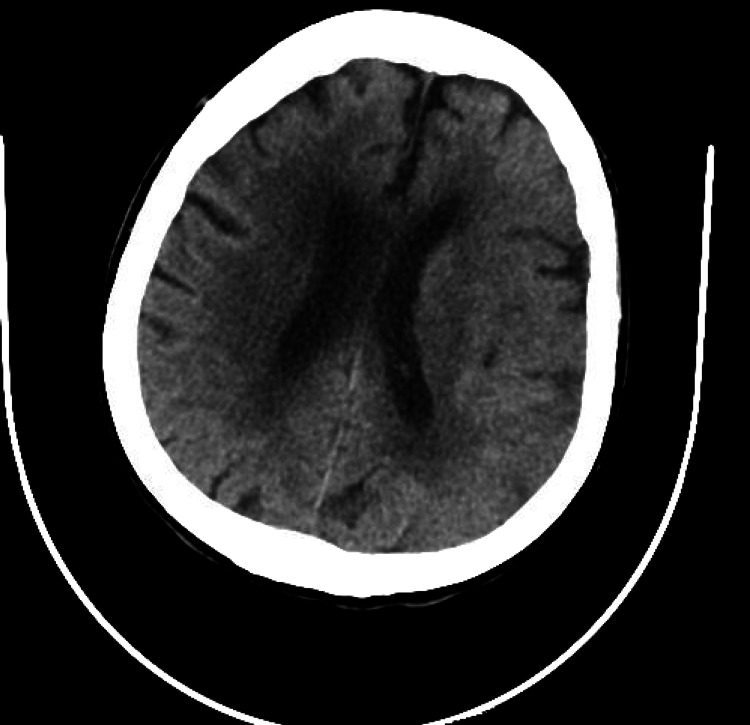
CT scan of the head negative for intracranial bleed or acute infarct.

In the ED, she had a blood pressure of 134/87 mmHg, pulse of 141 beats per minute, respiratory rate of 26, and oxygen saturation of 96% on room air. Physical examination revealed complete paralysis of the right upper and lower extremities and aphasia with a National Institutes of Health Stroke Scale (NIHSS) of 22. An electrocardiogram (EKG) showed AF with a heart rate of 116. CT scan of the head showed no evidence of acute infarct, acute intracranial hemorrhage, or mass effect, as showed in Figure 1.

**Figure 2:**
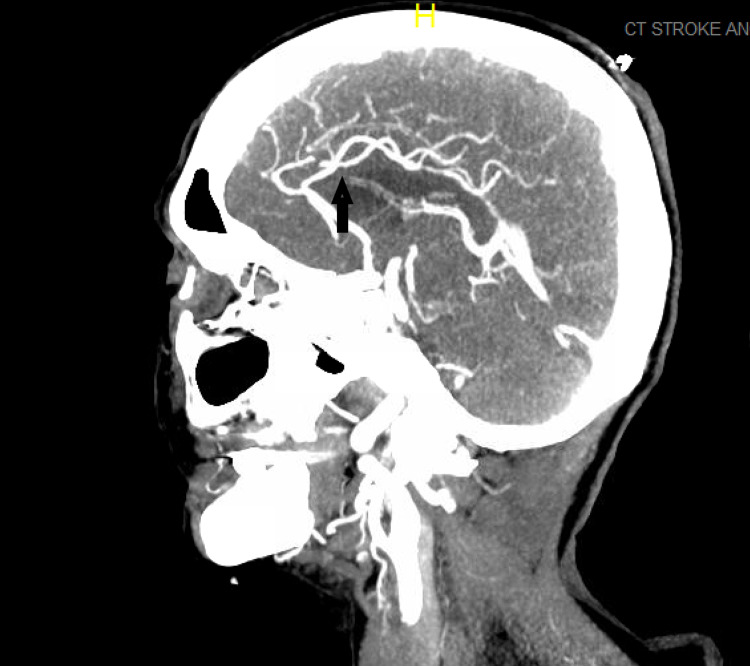
CT angiogram of head and neck showing the sagittal view of the middle cerebral artery occlusion.

Still, a CT angiogram showed complete occlusion of the M1 branch of the left middle cerebral artery, as shown in Figure 2.

Fibrinolytic therapy was withheld as she was on anticoagulation therapy for AF and had taken a dose less than 12 hours before her symptoms developed. She immediately underwent a successful mechanical thrombectomy and was transferred to the ICU for hemodynamic monitoring. A repeat CT scan 24 hours after her symptom onset was negative for bleed and ischemia. At that time, she regained most of the power in her right lower extremity and some of the strength in her right upper extremity, but her speech remained impaired. Gradually, her speaking and swallowing improved; she was started on physical therapy and discharged to a long-term rehabilitation center for continued physical therapy. She was discharged to the facility only on a half dose of apixaban for secondary prevention of stroke; the managing team discontinued aspirin and clopidogrel during the hospital stay because of the increased risk of bleeding.

## Discussion

Atrial fibrillation is an independent risk factor for CVA, and patients who are newly diagnosed with AF are typically started on anticoagulation therapy if their CHA2DS2-VASc score is higher than one and if there is an increased risk of bleeding [2]. Ischemic stroke is a consequence of a prothrombotic state resulting in occlusion of the cerebral arteries and is the most feared complication of AF. AF increases the risk of stroke by five-fold, and the use of anticoagulation reduces this risk by 60%, while single antiplatelet therapy reduces stroke by 20% [3]. More than 100,000 incidences of CVA that occur yearly in the United States are attributed to AF, and 40% of all strokes in patients >80 years of age are related to AF [4].

Other risk factors associated with stroke include hypertension, heart failure, diabetes, coronary artery disease, female sex, and advanced age, all of which convey an increased risk [5]. Our patient had a few of these risk factors, including advanced age, female sex, history of the previous stroke, peripheral vascular disease, in addition to the presence of AF, placing her at an exceedingly high risk of developing a stroke. She had a CHA2DS2-VASc score of seven points with an 11.2% risk of stroke per year. According to reports from her son, to mitigate these risks, she was prescribed aspirin, clopidogrel, and rivaroxaban, with which she was very compliant. 

Many trials have revealed that anticoagulation therapy in preventing stroke in patients with AF was superior to dual antiplatelet therapy (DAPT) in preventing stroke in this set of patients. A study reviewing The Atrial Fibrillation Clopidogrel Trial With Irbesartan for Prevention of Vascular Events (ACTIVE-W) trial showed that for patients with CHADS2=1, stroke rates were: 1.25% per year for patients receiving DAPT, specifically aspirin and clopidogrel and 0.43% per year for patients receiving anticoagulation therapy specifically warfarin (relative risk, RR=2.96, 95% confidence interval, CI: 1.26-6.98, p=0.01). For patients with a CHADS2>1, stroke rates were 3.15% per year and 2.01% per year, respectively (RR=1.58, 95% CI: 1.11-2.24, p=0.01). The benefits of oral anticoagulation (OAC) were not significantly different between these two groups, based on the CHADS2 score (p for interaction=0.19). However, a somewhat more significant absolute reduction in stroke among patients with CHADS2>1 than in patients with CHADS2=1 (1.14% per year versus 0.82% per year). The proportion of mild strokes (Modified Rankin 0-2) to severe strokes (Modified Rankin 3-6) was similar in patients with CHADS2=1 and >1 [5]. Later trials conducted to evaluate the use of direct-acting oral anticoagulants to warfarin in the prevention of stroke rates have proven non-inferiority; rivaroxaban versus warfarin in non-valvular AF (ROCKET AF) [6] and apixaban versus warfarin in patients with AF (ARISTOTLE) [7] trials respectively.

Studies have also been conducted to assess the use of more intensive approaches in the reduction of strokes. The TARDIS trial, done to evaluate the benefit of triple antiplatelet therapy versus the use of double antiplatelet therapy, showed no increased benefit as the incidence and severity of recurrent strokes and transient ischaemic attack (TIA) did not differ between the two therapies [8]. Patient outcomes from The Outcomes Registry For Better Informed Treatment Of Atrial Fibrillation (ORBIT-AF) registry were reviewed in a retrospective study by Steinberg et al. to assess whether aspirin and OAC are used compared to OAC therapy alone was associated with increased risks. This study demonstrated an increase in bleeding risks and hospitalization rates at six months with possible benefits in patients with coronary artery disease (CAD) history. However, there was not enough substantial evidence to recommend such therapy in patients with AF in reducing thromboembolic events [9]. Nonetheless, our literature search found no reported incidence of triple therapy failure in preventing a stroke hence making our case report unique and the first to be reported describing a case of failure of triple therapy even though aspirin and clopidogrel were prescribed for her peripheral vascular disease and only rivaroxaban was prescribed for the prevention of stroke given her history of AF.

For many years, guidelines on the management of AF had no inclination towards rate control or rhythm control. This may have been primarily based on the results of the AF follow-up investigation of rhythm management (AFFIRM) study in 2002 [10]. However, more recently, in 2020, the early rhythm-control therapy in patients with AF (EAST-AFNET 4) trial, demonstrated that a first-primary-outcome event occurred in 249 of patients assigned to early rhythm control (3.9 per 100 person-years) versus 316 patients assigned to usual care (5.0 per 100 person-years) with a hazard ratio, 0.79; and 96% CI, 0.66-0.94; p=0.005 [11]. Thus, it is now postulated that early rhythm control is associated with lower risks of adverse cardiovascular outcomes than usual care among patients with early AF and cardiovascular diseases. This recent discovery poses whether our patient’s cerebrovascular incident was due to being inadequately rhythm or rate controlled despite being on triple therapy. 

## Conclusions

It is unclear whether our patient’s CVA, despite taking aspirin, clopidogrel, and rivaroxaban, is due to clopidogrel or rivaroxaban not being as effective as other medications in the same classes or whether there was a confounding factor such as inadequate rate/rhythm control was involved. A review of our patient’s previous vital signs from hospital admissions and office visits showed that her rate was controlled, but on the day of presentation, her heart rate was in 140 s. Thus, one can argue that our patient was neither rhythm controlled nor rate controlled, and anticoagulation did not help prevent a stroke. As suggested by the more recent EAST-AFNET 4 trial, rhythm control was superior to rate control in patients with AF. Triple therapy reduces the risk of stroke with an acceptable increased risk of bleeding; however, there is no reported literature on the failure of triple therapy to prevent stroke. More studies are required to evaluate intensive approaches further, even possibly adding a fourth medication or procedures that can be done to avert CVA in high-risk patients with AF.
